# Enhancing in search of Milankovitch cycles from stratigraphic record using convex optimization algorithm

**DOI:** 10.1038/s41598-024-82720-0

**Published:** 2025-01-07

**Authors:** Syaiful Alam, Mohamad Sapari Dwi Hadian, Ahmad Helman Hamdani, Noorzamzarina Sulaiman

**Affiliations:** 1 Faculty of Geological Engineering, Universitas Padjadjaran, Sumedang, 45363 Indonesia; 2 Department of Geoscience, Faculty of Earth Science, Universiti Malaysia Kelantan, Campus Jeli, 17600 Jeli, Kelantan, Malaysia

**Keywords:** Milankovitch cycles, Stratigraphic record, Convex optimization, Palaeoclimate, Stratigraphy, Geophysics

## Abstract

Accurately identifying Milankovitch cycles has been a significant challenge in cyclostratigraphic studies, as it is essential for improving geochronology. This manuscript focuses on developing a method that distinguishes Milankovitch cycles from sedimentary noise to enhance stratigraphic precision. Despite their often-conspicuous magnitude, these periodicities frequently intertwine with noise, posing a challenge for conventional spectral analysis. Therefore, to address this issue, we have developed an algorithm that enhances the resolution of the Milankovitch signal by employing convex optimization in spectral analysis. To evaluate the effectiveness of this new algorithm, we applied it to four distinct types of local stratigraphy where the Milankovitch signal has been confirmed. These include the stratigraphic sections in the middle Miocene molluscan beds of Java and the Mahakam Delta, Pleistocene sediments of Hominin Flores, and the Towuti Lake in Sulawesi Island, Indonesia. Our findings demonstrate the preservation of all targeted signals, with a confidence level surpassing 99%. By setting the significance level to 1%, we can reject the null hypothesis, which assumes noise or the absence of a Milankovitch signal in the stratigraphic data being tested. The absence of deviations from the identified periodicities further strengthens the Milankovitch signal, underscoring the robustness of our algorithm. However, we acknowledge that achieving optimal results still hinges on the accurate selection of the initial parameters z and λ.

**Supplementary Information:**

The online version contains supplementary material available at 10.1038/s41598-024-82720-0.

## Introduction

Milankovitch signals are currently the focus of extensive research in the fields of climate stratigraphy, also known as cyclostratigraphy. The Milankovitch signals are characterized by periodicities of approximately 405-kyr, 100-kyr, 41-kyr, and 21-kyr, which are attributed to long- and short-eccentricity, obliquity, and precession, respectively^1–3^. In stratigraphic studies, the recognition of Milankovitch cycles plays an important role in the advancement of geochronology, particularly in the pursuit of high-resolution stratigraphy^4–6^.

Spectral analysis of stratigraphic records is an integral part of cyclostratigraphic studies. Stratigraphic records represent spatial time-series data, which can be analysed and statistically modelled in practice^7–9^. Autoregressive modelling is often employed to understand the patterns of allogenic and autogenic sedimentation cycles reflected in stratigraphic section. ^4,10^. The experiment we propose involves enhancing time-series data through convex optimization, enabling autoregressive modelling to estimate and recognize stratigraphic cycles most advantageously.

Using previously optimized Yule-Walker and Lomb-Scargle periodogram spectral analysis, we examined the capabilities of a combined approach. Although these two methods are distinct concepts, they complement each other effectively. Existing tools often have limitations in identifying Milankovitch signals and reducing noise in the stratigraphic record. Therefore, the comparative results we present in this article aim to address this gap. Adding this methodological framework could make recent algorithms better, making it easier to do more thorough study into the search for Milankovitch signals in cyclostratigraphic studies.

## Methods

The methodology of our study encompasses a series of systematic experiments designed to achieve primary outcomes, including noise reduction and signal enhancement in identifying Milankovitch cycles. The workflow initiates with convex optimization, where we configure initial matrices and variables essential for the autoregressive (AR) modeling process. By integrating the Yule-Walker AR model, we enhance the detection of Milankovitch signals, which are inherently related to sedimentation patterns observed in the stratigraphic record^11,12^.

### Convex optimization algorithm and parameter configuration

The implementation of convex optimization at the outset of this methodology effectively resolves Milankovitch signals with reduced noise compared to approaches without optimization. This algorithm represents a subfield of mathematics crucial for solving linear programming problems. Lithology serves as a fundamental dataset for our analysis, with each lithological variation numerically encoded as a prerequisite for time-series analysis.

We begin by setting the parameters z and λ, as well as the initial matrices A, B, C, and vector v, where the number of lithological data points is denoted as n, as shown in Eq. (1) to (6):

1 $$\:\lambda\:={10}^{-1}$$

2 $$\:z={10}^{-3}$$

3 $$\:A={\left[\begin{array}{cc}\begin{array}{cc}1&\:1\end{array}&\:\begin{array}{cc}1&\:\begin{array}{cc}\dots\:&\:1\end{array}\end{array}\end{array}\right]\:}_{1xn}$$

4 $$\:B={\left[\begin{array}{ccc}1&\:1&\:\begin{array}{cc}1&\:\begin{array}{cc}\dots\:&\:1\end{array}\end{array}\\\:-2&\:-2&\:\begin{array}{cc}-2&\:\begin{array}{cc}\dots\:&\:-2\end{array}\end{array}\\\:1&\:1&\:\begin{array}{cc}1&\:\begin{array}{cc}\dots\:&\:1\end{array}\end{array}\end{array}\right]}_{3xn}$$

5 $$\:C={\left[\begin{array}{ccc}1&\:0&\:\begin{array}{ccc}0&\:\dots\:&\:\begin{array}{ccc}0&\:0&\:0\end{array}\end{array}\\\:-2&\:1&\:\begin{array}{ccc}0&\:\dots\:&\:\begin{array}{ccc}0&\:0&\:0\end{array}\end{array}\\\:\begin{array}{c}0\\\:\begin{array}{c}0\\\:\begin{array}{c}\vdots \\\:\begin{array}{c}0\\\:\begin{array}{c}0\\\:0\end{array}\end{array}\end{array}\end{array}\end{array}&\:\begin{array}{c}-2\\\:\begin{array}{c}0\\\:\begin{array}{c}\vdots\\\:\begin{array}{c}0\\\:\begin{array}{c}0\\\:0\end{array}\end{array}\end{array}\end{array}\end{array}&\:\begin{array}{c}\begin{array}{ccc}1&\:\dots\:&\:\begin{array}{ccc}0&\:0&\:0\end{array}\end{array}\\\:\begin{array}{c}\begin{array}{ccc}-2&\:\dots\:&\:\begin{array}{ccc}0&\:0&\:0\end{array}\end{array}\\\:\begin{array}{c}\begin{array}{ccc}\vdots&\:\ddots\:&\:\begin{array}{ccc}\vdots&\:\vdots&\:\vdots\end{array}\end{array}\\\:\begin{array}{c}\begin{array}{ccc}0&\:\dots\:&\:\begin{array}{ccc}1&\:0&\:0\end{array}\end{array}\\\:\begin{array}{c}\begin{array}{ccc}0&\:\dots\:&\:\begin{array}{ccc}-2&\:1&\:0\end{array}\end{array}\\\:\begin{array}{ccc}0&\:\dots\:&\:\begin{array}{ccc}0&\:-2&\:1\end{array}\end{array}\end{array}\end{array}\end{array}\end{array}\end{array}\end{array}\right]}_{n-2xn}$$

6 $$\:v=\left[\text{0,1},2\right]$$

Matrix C is a tridiagonal matrix composed of components from matrices A and B, often referred to as a sparse matrix. Sparse matrices are used to efficiently represent large datasets with many zero elements, thereby reducing memory usage and computational cost. In this context, matrix A acts as an identity matrix, reflecting the direct contributions of observed values during the inversion process. Matrix B describes the relationships between observed values at neighboring locations in the inversion model^13,14^.

## Objective function and its components

Our optimization process revolves around a well-defined objective function, expressed as:

$$\:minimize\left\{z{\sum\:}_{i=1}^{n}{\left({litho}_{i}-{S}_{i}\right)}^{2}+\lambda\:{\sum\:}_{i=1}^{n-2}\left|{\left(C.S\right)}_{i}\right|\right\}$$

This function comprises two primary components: the first term quantifies the prediction error by calculating the squared differences between observed lithological data and the model’s predictions, while the second term, utilizing the L1 norm, encourages sparsity in the solution. The weights z and λ adjust the emphasis placed on fitting the observed data versus maintaining a parsimonious model. The effective balancing of these terms is crucial for achieving accurate and reliable identification of sedimentation cycles.

The first term of the objective function, $\:z{\sum\:}_{i=1}^{n}{\left({litho}_{i}-{S}_{i}\right)}^{2}$, quantifies this discrepancy through the sum of squared differences, emphasizing the importance of closely fitting the model to the observed data. A smaller prediction error indicates a more accurate representation of the lithological variations captured by the model.

Additionally, the second term, $\:\lambda\:{\sum\:}_{i=1}^{n-2}\left|{\left(C.S\right)}_{i}\right|$, introduces a regularization component that penalizes complexity in the solution. Here, C is the tridiagonal sparse matrix that we constructed, and the term​ $\:{\left(C.S\right)}_{i}$ accounts for the relationships between the predicted values in $\:S$ as dictated by the underlying sedimentation structure. This encourages sparsity in the solution, promoting a model that captures essential trends without overfitting to noise in the data.

## Sensitivity analysis

**Fig. 1:**
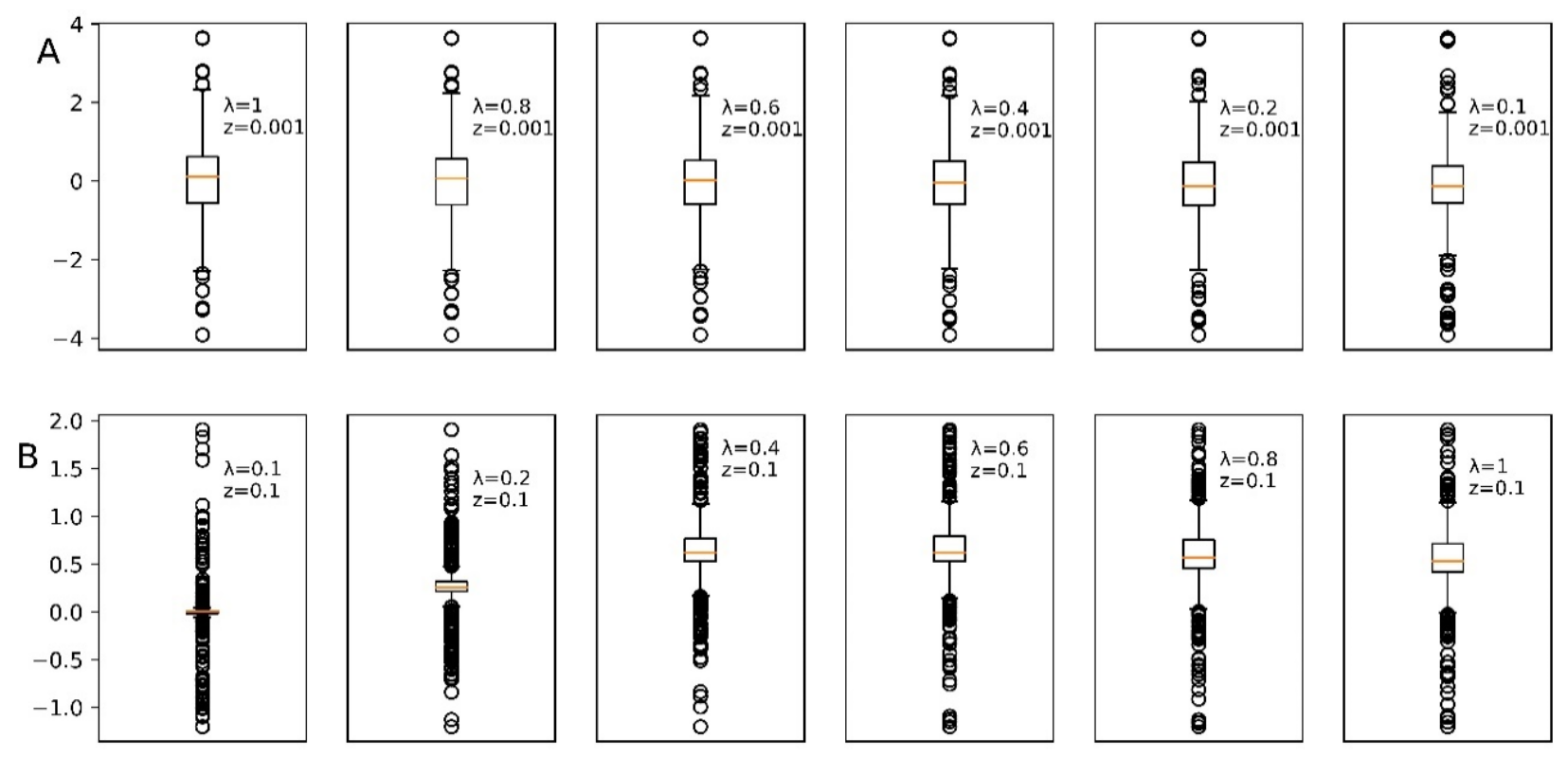
Boxplot of prediction error distribution using the Convex Optimized AR Model. (**A**) represents the error distribution resulting from varying λ with a constant z, while (**B**) stands for the opposite.

In this study, we conducted a sensitivity analysis to determine the impact of the parameters z and λ on the optimization results, considering that the selection of these parameters may appear arbitrary. Parameters z and λ are crucial in the algorithm we are testing. This analysis helps evaluate the effects of changes in these parameters, with the goal of minimizing prediction error. The boxplot presented in Fig. 1 illustrates the sensitivity to changes in parameters z and λ. In our case study, the optimal combination for z and λ is 0.001 and 0.1, respectively. This figure demonstrates that this combination results in a prediction error with a narrow interquartile range, and the outliers in the prediction error are not significant. Conversely, as the value of z increases, the boxplot for z = 0.1 with λ ranging from 0.1 to 1 shows numerous outliers in the prediction error. Under these conditions, the AR model struggles to effectively predict changes in data cycles due to the generation of significant prediction errors. The results of this sensitivity analysis underpin the selection of the z and λ values used in this study.

## Yule-Walker estimation

The subsequent step after obtaining the solution variables from convex optimization is the Yule-Walker estimation. We employed this estimation to understand the autoregressive relationships within the time-series dataset. Autoregressive coefficient values were obtained using maximum likelihood estimation to formulate the autoregressive equations^15,16^. This innovative aspect of our algorithm is of primary interest, as it computes residuals based on the results of convex optimization in autoregressive modeling^17–19^.

## Results

### Testing the algorithm on the synthetic cases

**Fig. 2:**
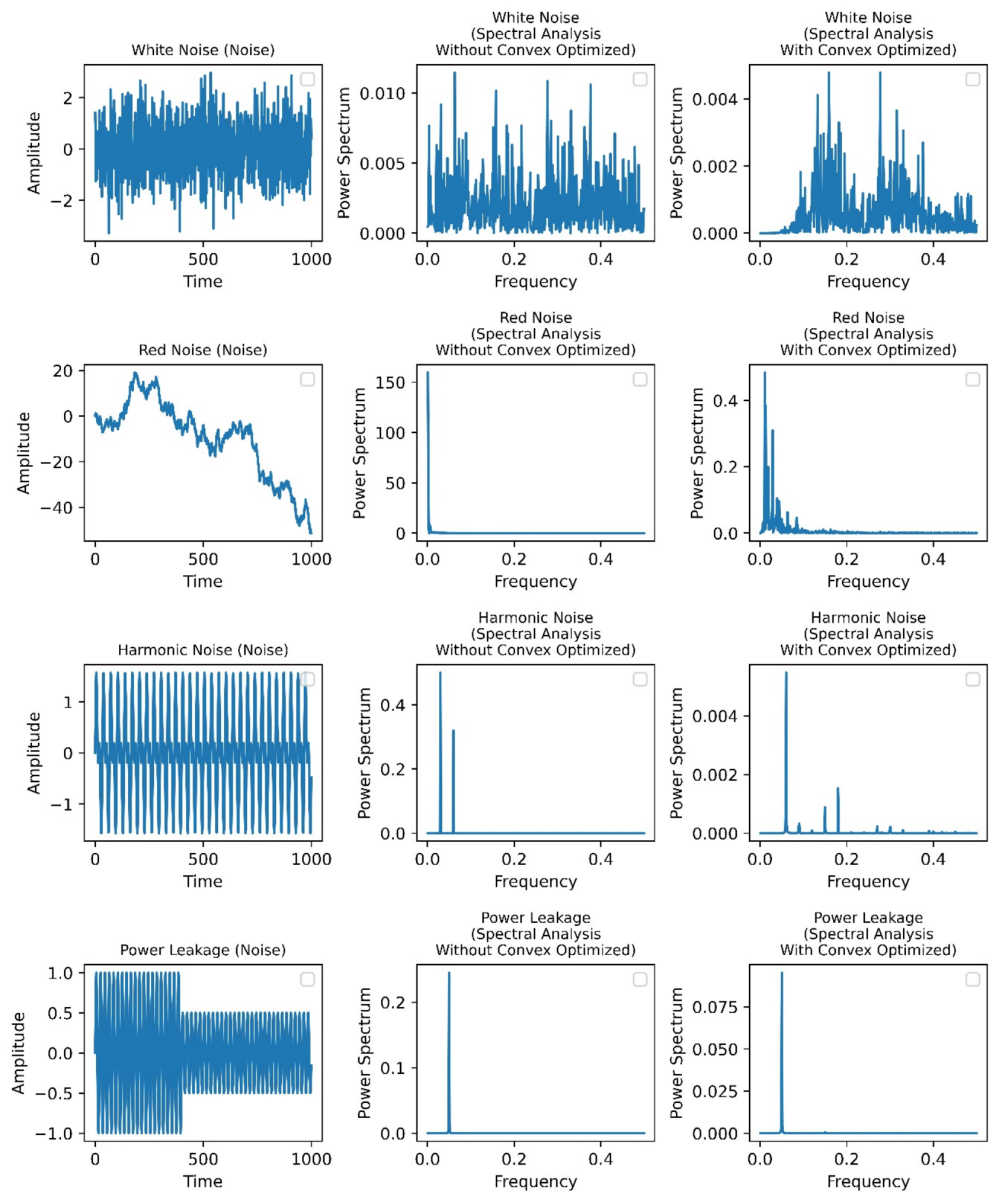
Comparison of spectral analysis of synthetic data (left), without (middle), and with convex optimization algorithm (right).

The convex optimization method we employed aims to enhance significant Milankovitch signals while mitigating the risk of inadvertently amplifying certain noise components. We meticulously designed the optimization function to focus on periodic signals, but further validation with synthetic datasets is essential to prevent artificial noise amplification. To evaluate the robustness of our algorithm, we generated synthetic time series incorporating target signals and various noise types, including red, white, harmonic noise, and power leakage. Testing indicated that our method effectively identifies Milankovitch cycles without amplifying noise, even under challenging conditions (see Fig. 2).

Unlike white noise, often overlooked in cyclostratigraphy, red noise, harmonic noise, and power leakage have gained attention due to their potential to obscure periodic signals^7,20^. Our synthetic data creation involved predetermined frequency properties to accurately evaluate the algorithm’s performance. Red noise, characterized by greater low-frequency energy, can obscure Milankovitch cycles, while harmonic noise may introduce artifacts affecting stratigraphic interpretation. Our convex optimization algorithm effectively sharpens frequency signals, particularly for organized noise types, while minimizing leakage into irrelevant frequencies. This advancement significantly enhances spectral analysis accuracy, facilitating the identification of relevant cyclical frequencies within stratigraphic data.

## Testing the algorithm on real cases

Our results have yielded promising outcomes from the algorithm tested on limited data from four locations across the islands of Indonesia (Java, Kalimantan, Sulawesi, and Flores) in relation to previously published cyclostratigraphic studies. Furthermore, Milankovitch signals from these four locations have been conclusively established. In this context, our algorithm is not expected to deviate from existing findings but rather to enhance and improve resolution in the search for Milankovitch signals. Lithological data from stratigraphic sections serve as inputs to the constructed algorithm.

**Fig. 3:**
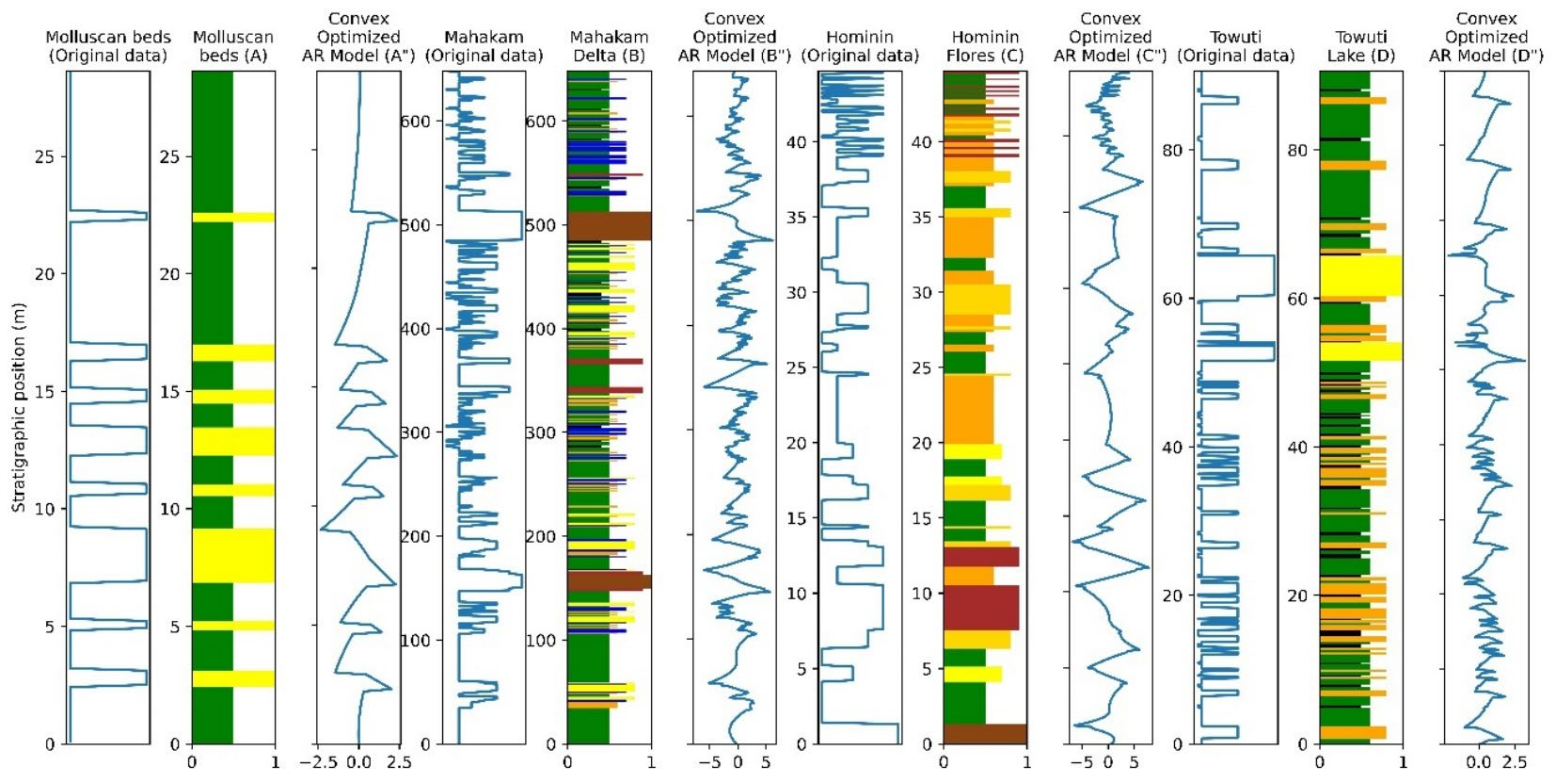
Stratigraphic Section and Convex Optimized AR Model. Molluscan beds is indicated and marked with (**A**) and their AR optimized model is represented as (A”), the Mahakam Delta stratigraphy is marked with (**B**) with its AR optimized model symbolized as (B”), the letters (**C**) and (C”) sequentially indicate the Hominin Flores section and its AR optimized model, while the Towuti Lake section is represented by (**D**) and (D”) for the AR optimized model result.

Figure 3 presents the stratigraphic sections from the four locations, along with the autoregressive (AR) model obtained through Yule-Walker estimation with an order of five after convex optimization. The color variations in the stratigraphic sections represent lithological diversity, with green denoting claystone, orange representing siltstone, yellow indicating sandstone, brown signifying gravel beds, black depicting peat layers, and blue reflecting limestone. All the stratigraphic sections have been confirmed to exhibit periodicities corresponding to Milankovitch signals. Consequently, through convex optimization, we aim to enhance signal resolution and suppress noise without introducing deviations in the spectral values of the Milankovitch signals.

## Molluscan beds, West Java

The Nyalindung Formation has been the subject of extensive research utilizing mollusks as indicators of sea-level changes, particularly known for its middle Miocene molluscan beds^21^. Studies have correlated the Cijarian riverbed section with the deeper facies within the Nyalindung Formation, suggesting an influence from Milankovitch cycles with a periodicity of approximately 41,000 years. This relationship between orbital changes and climate is supported by various findings, including the middle Miocene climatic optimum around 11.5 million years ago^22–26^, glacio-eustatic fluctuations exceeding ± 50 meters^27–30^, and mid-latitude temperatures that were 3–6 °C higher than present-day conditions^31,32^.

The Nyalindung Formation consists of alternating layers of sandstone and claystone with a total thickness of about 25 m, which create discernible sedimentary cycles (see Fig. 3). The stratigraphic section reveals eight distinct cycles, which are also evident in the Yule-Walker estimation results optimized through the convex optimization method. A detailed examination of the convex-optimized autoregressive (AR) model indicates a significant curvature at lithological boundaries, particularly around sandstone-claystone contacts. Conversely, the AR model curve exhibits a smoother profile within the claystone intervals, indicating its capacity to capture cyclical changes in the stratigraphic section.

The predictable cyclicity observed in the molluscan beds provides insights into more complex sedimentary systems. The Yule-Walker algorithm, foundational to linear forward prediction, demonstrates a cumulative sum of prediction error values within the convex-optimized AR model section. The molluscan beds from 0 to the maximum thickness allow us to analyze this method’s functionality. In lithologically homogeneous intervals, cumulative prediction errors tend to cancel each other out, maintaining values between 0 and 2 m in stratigraphic position, particularly evident at depths greater than 23 m. However, significant lithological differences between intervals result in noticeable deflections in the convex-optimized AR model curve, as observed between 3 and 17 m in stratigraphic position, highlighting the trend produced by lithological changes that do not counterbalance.

### Mahakam Delta, Kalimantan

The Mahakam Delta, located in eastern Borneo (Kalimantan Island), represents an intriguing sedimentary feature, encompassing both recent and ancient sediments. Despite its equatorial location, various internal and external factors have been identified as influences on the delta’s growth^33–35^. Notably, even in the absence of glaciers, warming and cooling events have significantly impacted the delta’s development, emphasizing the crucial role of climate in deltaic sedimentation^36,37^. The progradation cycles of the Mahakam Delta into the Makassar Strait, coupled with shifts in delta lobes, suggest that both internal dynamics and external climate controls shape coastal morphology^35,38,39^.

The variability of the middle Miocene Climatic Optimum (MMCO) is likely recorded in the sedimentary sections of the Mahakam Delta. The MMCO represents a brief period of warm climate change during a more extended phase of cooling since the last extreme warm climate at the Paleocene-Eocene boundary^31,40^. Within the Mahakam Delta, three sedimentation cycles in the Kunjang River reflect Milankovitch orbital cycle controls at varying scales. The preserved Milankovitch cycles in the deltaic record, particularly in the Kunjang riverbed section, encompass periods of 18.1–22.2 kyr, 41.7 kyr, and 94.5–125 kyr, with the obliquity signal being the most dominant^36^.

The Mahakam Delta section, approximately 650 m thick, reveals three long sedimentation cycles, categorized by stratigraphic meter positions of 0–150 m, 150–500 m, and 500–650 m. Sedimentation cycle boundaries are characterized by the presence of gravel beds, indicated by orange coloration in Fig. 3, at stratigraphic positions around 150 m and 500 m. The convex-optimized AR model effectively identifies these boundary changes, demonstrating greater curve deflection at these transition points compared to the shorter cycles within the three extended cycles.

### Hominin Fossil Strata, Flores

In addition to studies at the Sangiran site, numerous archaeological and geological investigations of Hominin fossils have been conducted in Flores, Indonesia^41–44^. Researchers have employed various age-dating methods at these sites, integrating geomagnetic polarities from the Matsuyama and Brunhes epochs with the stratigraphic sections containing Hominin fossils. These studies indicate that the Hominin fossil layer dates to approximately 0.781 million years ago, closely aligning with previous estimates of around 700 kyr^45^. However, cyclostratigraphic analyses at this site have yet to complement magnetostratigraphic studies, leaving the influence of orbital climate dynamics on the Hominin fossil record largely unexplored. The age constraints and sedimentation cycles within this section serve as a foundation for examining Milankovitch cycles affecting these Pleistocene sediments.

The Pleistocene sediments present an intriguing case, particularly due to a climate transition within this interval, shifting from 41-kyr obliquity to 100-kyr eccentricity in younger sediments. Evidence suggests correlate sediment cycle thickness ratios with Milankovitch cycle periodicities, noting that sedimentation cycles influenced by eccentricity typically exhibit thicker deposits compared to those governed by precession^2,3,46,47^. The convex-optimized AR model indicates that in the Hominin Fossils section, thicker sedimentation cycles are observed between 0 m and approximately 35 m, while thinner cycles are present above that depth. This correlation may result from the climate shift observed during the Pleistocene.

### Towuti Lake Sediment, Sulawesi

Among various study sites, Lake Towuti is located closest to the Indo-Pacific Warm Pool (IWP), an area characterized by above-average sea surface temperatures. Changes in temperature in this region have significant implications for global climate and marine ecosystems^48–50^. The sediments in Lake Towuti correspond to the Pleistocene, coinciding with Hominin fossil strata. Researchers at Towuti Lake have investigated paleoenvironmental conditions and geochronology by integrating borehole data and magnetic susceptibility studies. Their findings suggest that eccentricity-controlled sedimentation can be traced back approximately 772 kyr to 903 kyr^51^.

The convex-optimized AR model for the Towuti Lake section reveals two distinct frequency ranges between 0 m and approximately 45 m, and between 45 m and 90 m in stratigraphic position. These frequencies may indicate the mid-Pleistocene climate shift within this interval. In essence, the convex-optimized AR model effectively recognizes changes in sedimentation cycles, highlighting the dynamic geological history of the region.

### Spectral analysis

Spectral analysis is a widely used method for characterizing frequency and periodicity in geological time-series data. Several techniques have been developed for spectral analysis, including the periodogram, Blackman-Tukey method, Maximum Entropy method, and Multi-Taper method^52,53^. The periodogram, in particular, is effective for analyzing lithological data, which typically exhibits minimal noise. To address irregular sampling intervals in our dataset, we employed the Lomb-Scargle algorithm, offering a robust alternative to the classical Fast Fourier Transform periodogram.

**Fig. 4:**
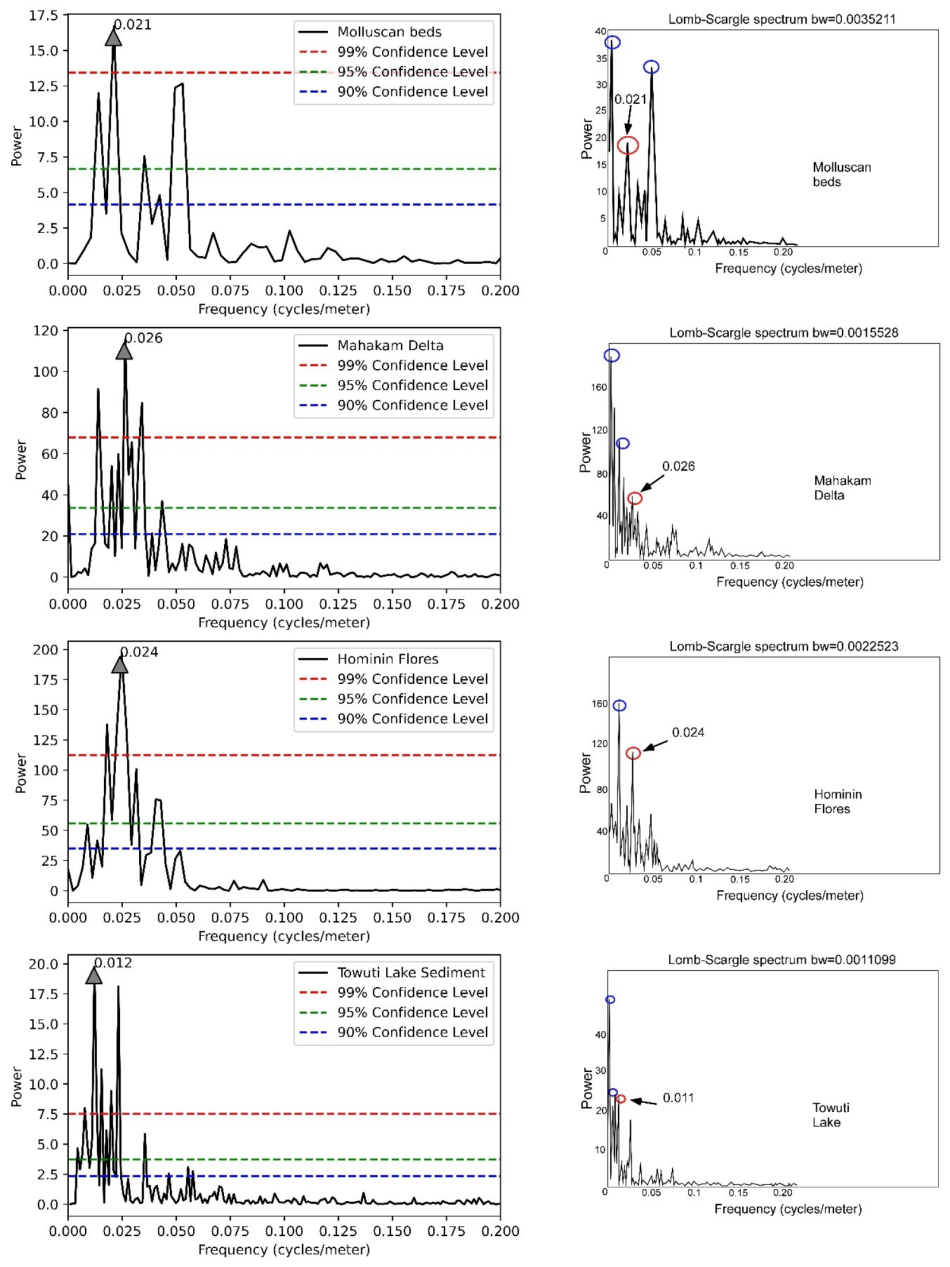
(Left) represents the spectral analysis result of Molluscan beds, Mahakam Delta, Hominin Flores, and Towuti Lake Sediment optimized through the convex algorithm, with a gray arrow symbol indicating the peak of the reference target frequency. (Right) The spectral analysis using ACycle. The red circle represents the reference target frequency, while other peak frequencies are indicated by blue circle, which are not the reference target signal.

To establish confidence levels, we utilized the chi-square distribution, which helps determine the threshold distinguishing significant signals from noise. We calculated critical chi-square values for confidence levels of 99%, 95%, and 90%, based on a specified degree of freedom (phi). In our Fourier analysis, these confidence levels are depicted as horizontal lines on the power spectrum, indicating thresholds above which signals are considered significant (Fig. 4). Signals exceeding these thresholds are interpreted as meaningful Milankovitch signals, while those below are classified as noise. Narrow confidence intervals provide greater precision in detecting significant signals but may overlook subtle data characteristics, while wider intervals capture more variability at the cost of precision. By applying various confidence levels, we ensure that our spectral analysis results are robust, balancing precision and inclusivity in interpreting Milankovitch signals.

We evaluated the effectiveness of the Lomb-Scargle algorithm by assessing its capability to resolve Milankovitch signals through spectral analysis, aligning with findings from four previous studies: the Molluscan beds, Mahakam Delta, Hominin Flores, and Towuti Lake sediment. The Molluscan beds, Mahakam Delta, and Hominin Flores exhibit a dominant obliquity signal characterized by a periodicity of approximately 41,000 years, while Towuti Lake sediments are primarily influenced by eccentricity signals, predicting a periodicity of about 105,000 years. Importantly, all spectral analyses were conducted on time-series data that had undergone convex optimization.

Our analysis revealed a dominant frequency of 0.021 cycles per meter in the molluscan beds, corresponding to a spectral density of approximately 47.6 m per cycle, closely aligning with the obliquity periodicity. The detected spectral density of 47.6 m, nearly double the total thickness of the stratigraphic section (28.9 m), suggests that the stratigraphic record captures only a portion of the full sedimentary cycle. This indicates the potential existence of a longer-term depositional cycle that may not be fully represented in the available section, possibly due to incomplete deposition, erosion, or stratigraphic hiatus. The observed frequency may also reflect spectral leakage or aliasing effects resulting from limited stratigraphic coverage.

Within the context of the middle Miocene deposition age of the Nyalindung Formation, this biozone falls within N13^54,55^, characterized by the last appearances of *Fohsella fohsi* around 11.79 Ma and *Globigerinoides subquadratus* around 11.63 Ma^56^, spanning approximately 160,000 years. The presence of four depositional sequences within the Nyalindung Formation, termed deepening-shallowing facies, along with the dominant 47.6-meter cycle associated with obliquity, suggests a deposition duration of approximately 190,000 years. This estimate closely approximates the range of biozone N13^21^, derived from the dominant frequency indicating a depositional cycle of about 47.6 m, leading to an estimated total sediment thickness of around 190 m when considering four depositional cycles. Although this estimate is subject to debate, several studies have utilized a linear ratio between cycle thickness and eccentricity cycle parameters to support this interpretation, aligning with the Sadler effect, which assumes minimal hiatuses and a relatively high sedimentation rate in the Nyalindung section, particularly given its thin total section measuring less than 50 m.

Geomagnetic polarity and biostratigraphy further illuminate the geological and climatic history of the study area. Climate records can be inferred from the distribution of δ^18^O. The most relevant analysis to the Mahakam Delta occurs within the Asia-Pacific Region^39,57–59^, indicating that sediments from the Mahakam Delta were formed between 13.7 Ma and 14.6 Ma during the middle Miocene. The obliquity imprint is evident along the C5ADn chron at the Sungai Kunjang site within the Mahakam Delta section^36^, spanning stratigraphic positions from 0 to 350 m. According to argon dating, the C5ADn polarity chron has an age range of 15.12 Ma to 15.66 Ma, lasting approximately 540,000 years^57,60^. The convex-optimized autoregressive model identified 11 to 13 cycles within this stratigraphic position, revealing a strong obliquity signal spectrum with a frequency of 0.026 cycles per meter in the Lomb-Scargle periodogram. These findings suggest that sediments were deposited over a timeframe of 423,500 to 500,500 years, corroborating the existing geomagnetic polarity timescale for this interval. Although the obliquity period resolved by the convex optimization algorithm is 38.5 m per cycle (or 0.026 cycles per meter), this value remains consistent with the obliquity range of 35.3 to 41.5 thousand years^36^.

In the Hominin fossil section, spectral analysis reveals a strong frequency of 0.024 cycles per meter, corresponding to a period of approximately 41.7 m per cycle, closely aligned with the ~ 41,000-year obliquity periodicity. Despite this strong signal, the cycle thickness ratio between this frequency and the 0.01 cycles per meter frequency (with a period of ~ 100 m per cycle) differs slightly from the expected 20:5:2:1 Milankovitch cycles ratio among eccentricity, obliquity, and precession cycles^2,3,46,47^. To address this discrepancy, it is important to consider that variations in sedimentation rates within this section may compress the obliquity cycle, thus affecting the observed thickness ratio. This phenomenon can be explained by the Sadler effect^61^, wherein shorter cycles, such as those related to obliquity, are more susceptible to compression in the sedimentary record.

In the Towuti Lake section during the mid-Pleistocene interval, the dominant frequency is 0.012 cycles per meter, corresponding to a period of approximately 83.3 m per cycle, along with a frequency of 0.023 cycles per meter, which produces a period of 43.5 m per cycle. These frequencies potentially reflect eccentricity and obliquity cycles; however, the ratio between these two frequencies is lower than the theoretical value for short eccentricity versus obliquity cycles. This discrepancy can also be elucidated by the Sadler effect, where the longer eccentricity cycle may influence sedimentation variations, causing the obliquity cycle to appear more prominent. The use of the convex-optimized autoregressive model algorithm enhances the obliquity signal, demonstrating that global climatic influences can still be detected across these distinct locations, despite non-ideal cycle thickness ratios.

Our analysis underscores the importance of interpreting signals from spectral analysis to avoid misleading conclusions. High-magnitude frequency peaks may indicate dominant signals in stratigraphic data but can also represent noise. Conventional spectral analysis without convex optimization often produces significant noise surrounding the expected spectral peaks. Thus, a comprehensive interpretation of sedimentary signals is essential for accurately identifying target frequencies.

**Fig. 5:**
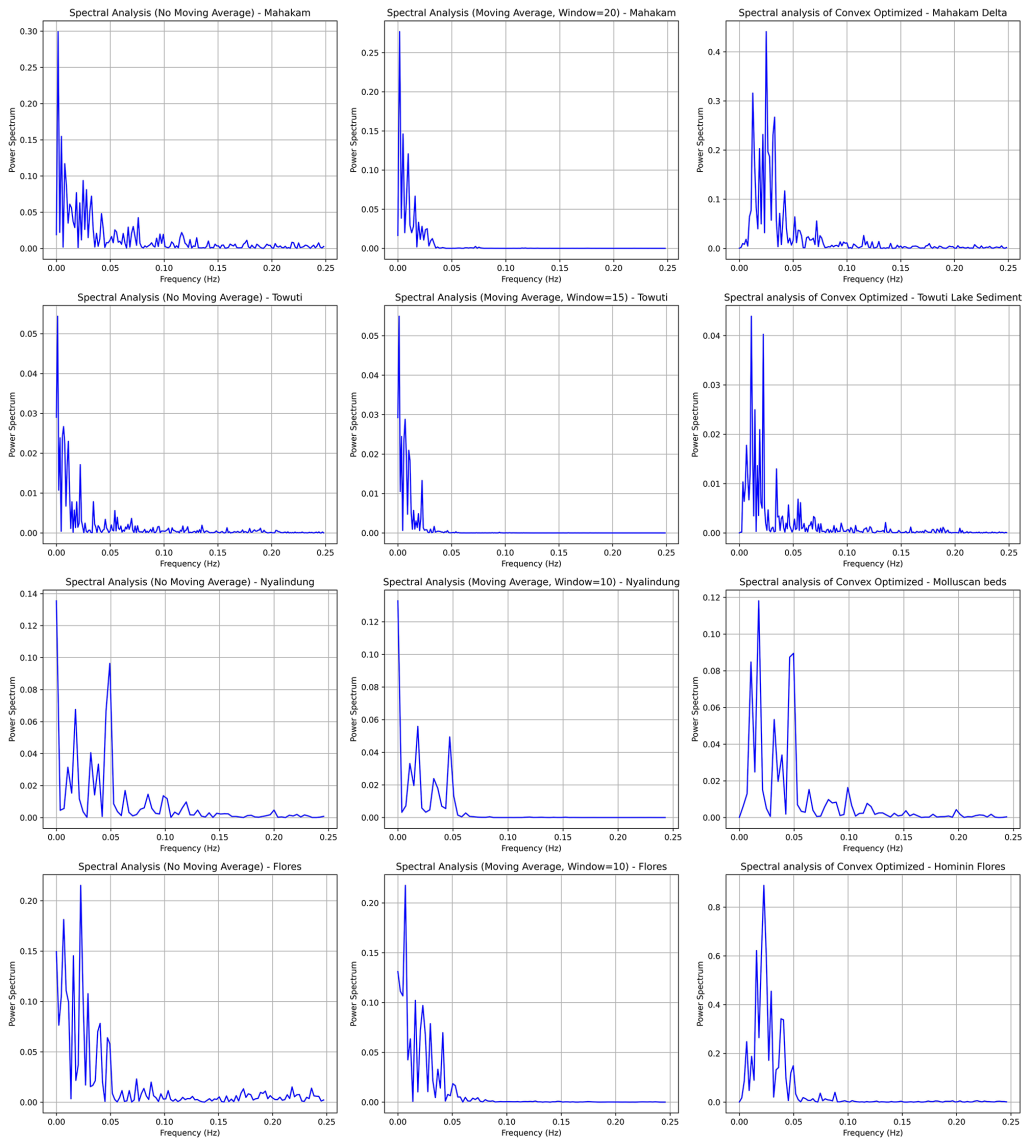
Comparison of raw data spectral analysis, smoothed data spectral analysis, and spectral analysis of data processed through the convex optimization algorithm.

Conducting spectral analysis on raw, smoothed, and convex-optimized data is vital for understanding embedded signal characteristics (Fig. 5). Raw data analysis enables direct signal identification, though noise often complicates the detection of Milankovitch cycles. Data smoothing techniques, such as moving averages, reduce noise but may obscure critical information. Convex optimization, as a subsequent step, offers better noise reduction while preserving key spectral features. Comparing these approaches enhances our understanding of each method’s contributions to improving signal detection and establishing optimal conditions for interpreting stratigraphic data.

**Table 1:** Comparison of frequency, power, and period.

Section	Convex Optimized AR Model			ACycle Software		
	Frequency	Power	Period	Frequency	Power	Period
Molluscan beds	0.021	16.71	47.6	0.021	19.26	47.6
Mahakam delta	0.026	115.47	38.5	0.026	60.4	38.5
Hominin Flores	0.024	196.93	41.7	0.024	113.5	41.7
Towuti Lake	0.012	19.75	83.3	0.011	23.6	90.9

## Discussion

Detecting Milankovitch cycles is fundamental to cyclostratigraphic studies. When sedimentary cycles in a stratigraphic section align with Milankovitch cycles on a kiloyear (kyr) scale, it allows for high-resolution geochronology and stratigraphy. These cycles are advantageous due to their distinct periodicities of 405 kyr, 100 kyr, 41 kyr, and 26 kyr. However, various noise sources, including autogenic and allogenic controls, may obscure these periodicities in the stratigraphic record^10,62,63^. Nevertheless, spectral analysis often uncovers many of these signals.

Currently, several software tools are being developed to detect Milankovitch signals from stratigraphic data, with Astrochron and ACycle being among the most prominent^64–66^. Astrochron employs R for statistical analysis, while ACycle utilizes Matlab. Our study specifically compares our methodology with ACycle, as it includes a Lomb-Scargle periodogram feature essential for spectral analysis. Our approach distinguishes itself through the inclusion of a convex optimization process.

Table 1 summarizes the results of Milankovitch spectral density detection obtained through our algorithm and compares them with ACycle. Both the convex optimized autoregressive (AR) model and ACycle yield similar spectral density values, with the former enhancing the signal spectrum while minimizing noise. Although both methodologies produce comparable frequency and period values, discrepancies in power values can occur. However, these power values are less critical in practical terms as long as they effectively differentiate signal from noise. Our spectral analysis primarily focuses on frequency values to relate them to the periodic nature of the Milankovitch signal.

Variability in signal amplitude within stratigraphic records can be influenced by sedimentation rate fluctuations^4^. Changes in signal amplitude are complex and often associated with astronomical cycles identified by Milankovitch. Records with smaller stratigraphic intervals (less than 100 m) can capture climate cycle variations. The continuity of sedimentation across astronomical timescales may be affected by local environmental factors such as subsidence and sedimentation processes. Examples from various regions and geological epochs (Neogene, Mesozoic, Pleistocene) demonstrate the influence of Milankovitch cycles on stratigraphic patterns^4^. Notably, even thin layers, such as the approximately 25-meter-thick Molluscan beds in West Java, and thicker deposits like those in the Mahakam Delta (650 m), reveal complex cycle patterns and highlight the significant impact of astronomical cycles on sedimentation.

### Application in other stratigraphic studies and IODP Data

**Fig. 6:**
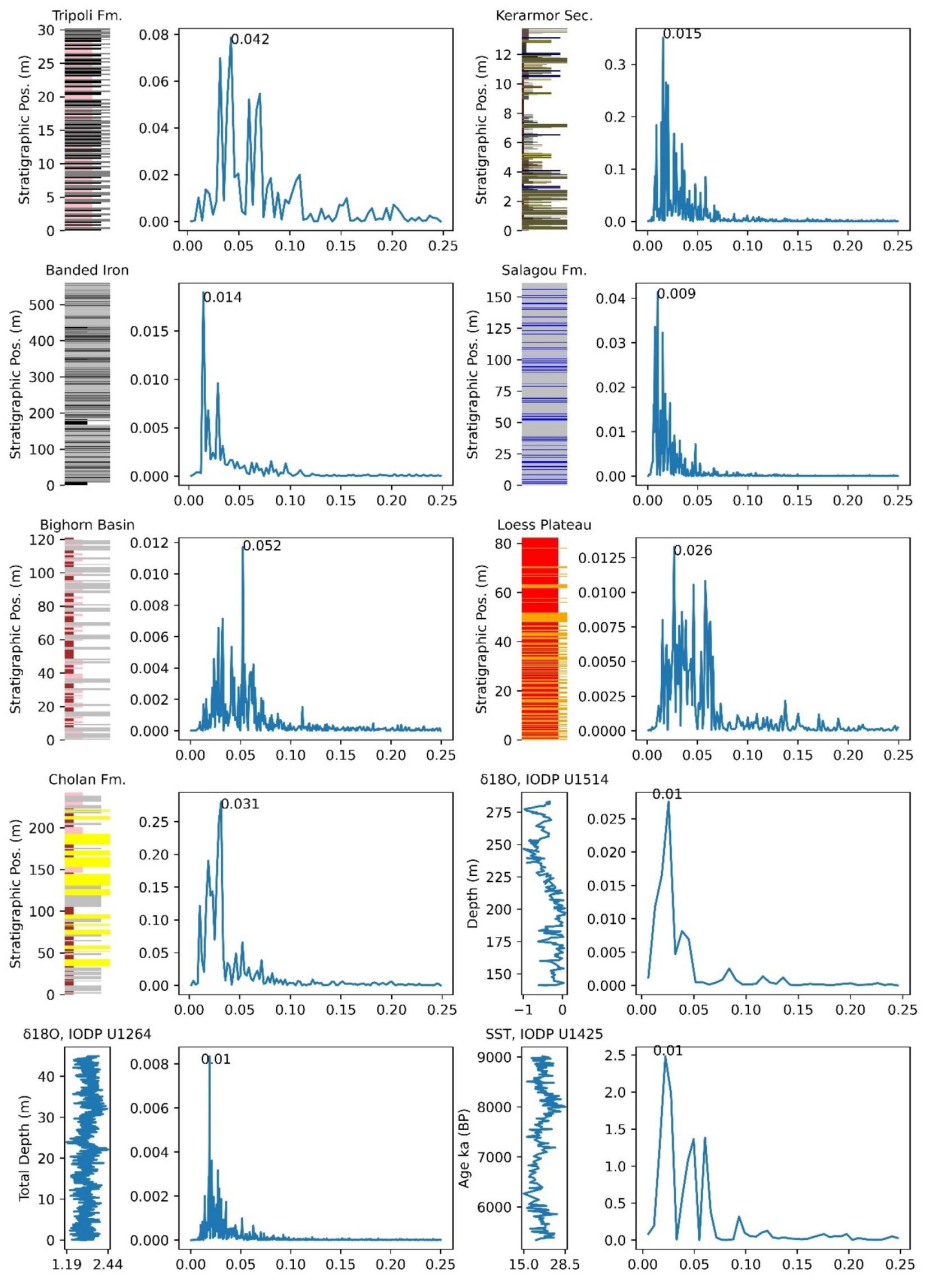
Stratigraphy and their corresponding spectral analysis results. The dominant frequencies in the Tripoli Fm., Banded Iron, Bighorn Basin, Cholan Fm., Keramor sec., Salagou Fm., and Chinese Loess Plateau are respectively 0.042, 0.014, 0.052, 0.031, 0.015, 0.009, and 0.026, with a peak frequency at 0.01 from δ^18^O of IODP U1514, δ^18^O of IODP U1264, and Sea Surface Temperature (SST) of IODP U1425.

The convex optimized autoregressive (AR) model algorithm has proven effective in analyzing various stratigraphic sections, including the Tripoli Formation^67^, banded iron formations, BIF^68^, Willwood Formation in the Bighorn Basin^69^, Cholan Formation^70^, Kerarmor Member of the Postolonnec Formation^71^, Salagou Formation^72^, and the Chinese Loess Plateau^73^. It has also been applied to δ^18^O data from IODP Sites U1514^74^ and U1264^75^, as well as sea surface temperature data from IODP Site U1425^76^. Previous studies identified Milankovitch cycles in these units, establishing a framework to test the algorithm’s robustness and effectiveness in detecting Milankovitch signals across diverse environments. The stratigraphic thicknesses analyzed range from tens to hundreds of meters, encompassing various lithological compositions and sedimentation patterns influenced by eccentricity, obliquity, and precession factors. Results are summarized in Fig. 6.

In Fig. 6, dominant cycles are linked to the precession cycle in the Tripoli and Willwood Formations, the eccentricity cycle in the BIFs, Kerarmor Member, and Salagou Formation, and the obliquity cycle in the Cholan Formation and Chinese Loess Plateau. The Milankovitch cycles identified through this convex optimization do not deviate from prior findings but show improved detection of the dominant frequencies affecting stratigraphic cycles.

Milankovitch cycles, particularly the precession cycle, significantly influence climate conditions and sedimentation patterns in the Tripoli and Willwood Formations. In the Tripoli Formation, the precession cycle drives ‘dry-wet’ oscillations, affecting water column stratification and sediment deposition, ultimately facilitating diatomite and sapropel formation. In the lower Eocene Willwood Formation, the precession cycle predominates, with a cycle thickness of approximately 7.1 m correlating with a 21.6-thousand-year period, influencing sedimentation during phases of floodplain stability and avulsion.

The Cholan Formation of Taiwan and the Chinese Loess Plateau also exhibit significant impacts from Milankovitch cycles. The dominant obliquity cycle in the Cholan Formation correlates with sea-level fluctuations linked to changes in summer insolation in the Northern Hemisphere during the early Pleistocene. Correlation with climate curves, such as δ^18^O, reveals strong similarities with deposition cycles. Furthermore, the stratigraphy of the Cholan Formation offers archaeological evidence of Milankovitch cycle influences on sea-level changes and deposition in Southeast Asia during the early Pleistocene. The obliquity cycle governs summer monsoon variations in the eastern Chinese Loess Plateau, supported by Rb/Sr analysis of red clay layers in Shilou. Deep-sea oxygen isotope records indicate that the obliquity cycle dominated global climate before the mid-Pleistocene climate transition, aligning with findings in the Chinese Loess Plateau.

In banded iron formations, particularly within the Joffre and Kerarmor Members, cyclostratigraphic analyses indicate consistent lithological variations. In the BIFs, layer alternations occur at approximately 85 cm intervals, linked to short eccentricity cycles, while larger cycles measuring 3.3 m correspond to long eccentricity cycles. In the Kerarmor section, eccentricity cycles correlate with thicknesses of about 2.5 to 4 m, suggesting high sedimentation rates and potential long-term deposition compression.

A study at IODP Site U1425 in the Japan Sea highlights the dominant Milankovitch cycle as eccentricity, with significant peaks in short eccentricity cycles observed at a frequency of approximately 0.01, refining the orbital age model for this site and enhancing insights into paleoceanographic conditions. Similarly, at IODP Site U1264, the eccentricity cycle emerges as the predominant component, particularly at 110-kyr and 405-kyr periods. The 110-kyr short eccentricity cycle is evident in benthic stable isotope (δ^18^O and δ^13^C) and CaCO₃ records, influencing climate changes and the Earth’s cryospheric system during the Oligocene-Miocene period. At Site U1514, the eccentricity cycle is recognized as the primary driver of rhythmic variations in darker layers, influencing lithological properties and Ca/Fe content.

Despite the Kerarmor section’s limited stratigraphic thickness of 14 m, Milankovitch cycle signals remain detectable. This suggests that high sedimentation rates can mitigate the Sadler effect, which posits that sedimentation rates decrease over extended observation periods due to hiatuses. High sedimentation rates facilitate a more continuous stratigraphic record, allowing clearer identification of climate cycles spanning tens to hundreds of thousands of years.

### Evaluation against background noise

Distinguishing the types of noise in stratigraphic records is crucial, as different noise types can impact Milankovitch signal detection. Autocorrelation analysis (ACF) categorizes stratigraphic time series by their noise levels. White noise exhibits negligible autocorrelation, while red noise shows strong positive autocorrelation at small lags that gradually diminishes. Convex optimization algorithms must be evaluated for their effectiveness in enhancing the signal-to-noise ratio, especially in the presence of red noise.

**Fig. 7:**
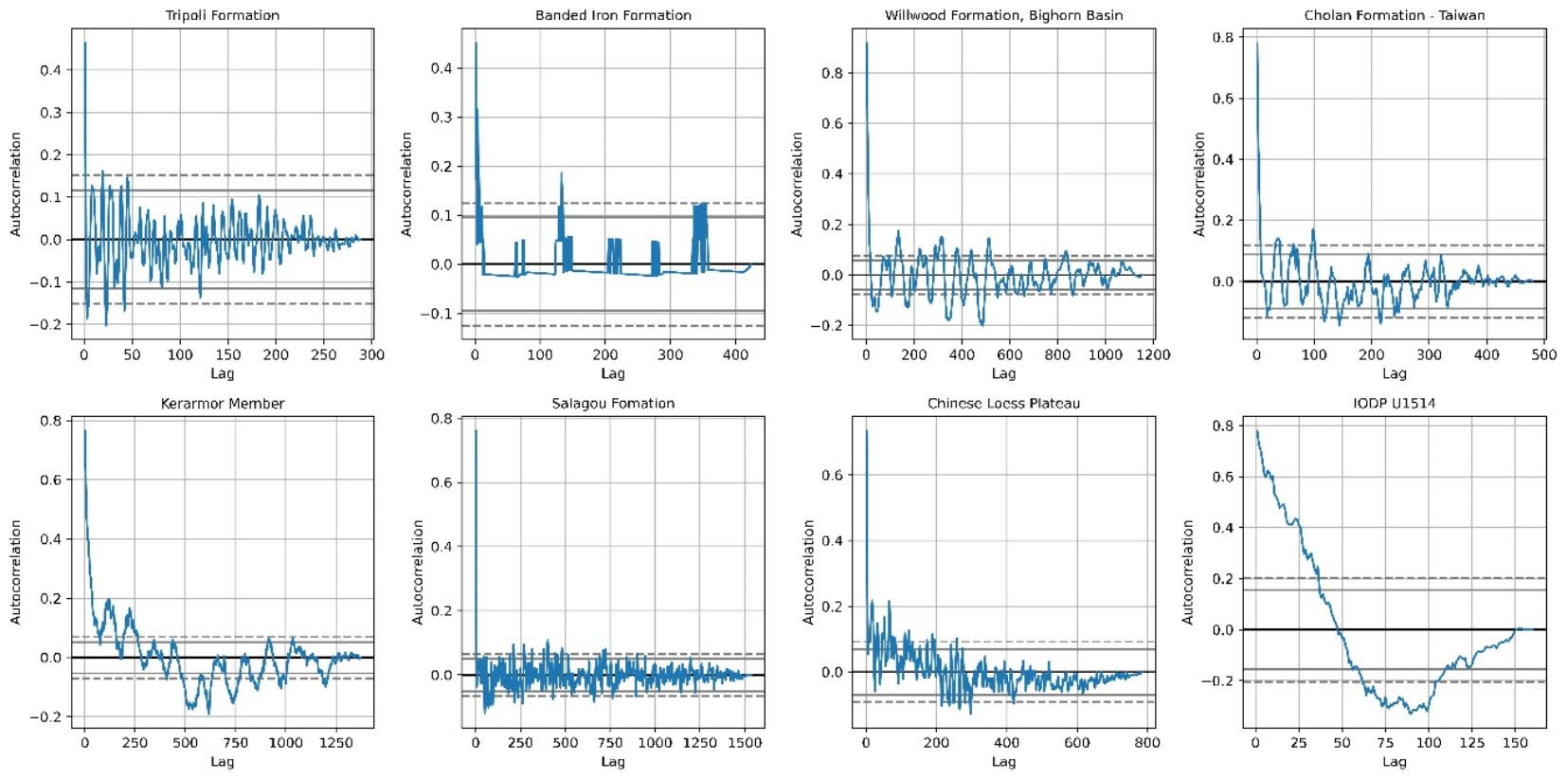
Autocorrelation of the stratigraphic data series used for testing the convex optimization algorithm.

The autocorrelation subplots in Fig. 7 display various noise types across different data series. Red noise is evident in formations such as the Banded Iron, Kerarmor Member, and several IODP datasets, indicating climatic cycles’ strong influence on sedimentary processes. Harmonic noise appears in data from the Willwood Formation and Chinese Loess Plateau, suggesting periodic signals possibly linked to Milankovitch cycles. Power leakage may occur in formations like Salagou, where strong signals dissipate with residual fluctuations, indicating high-frequency signals leaking into lower frequency spectra. Conversely, white noise is evident in the Tripoli and Cholan Formations, where no significant autocorrelation patterns indicate random noise.

In contemporary sedimentology research, red noise, harmonic noise, and power leakage are significant concerns, reflecting the interactions between autogenic and allogenic sedimentation processes. Identifying these noise types is essential for understanding sedimentation dynamics and revealing underlying climatic signals. The convex optimization algorithm shows promise in effectively functioning across various noise backgrounds, thereby enhancing the reliability of Milankovitch cycle detection.

## Conclusion

A comparative analysis was conducted between our convex optimization algorithm and the ACycle method, which is widely used in cyclostratigraphic studies. The spectrograms generated by both methods were compared using both real and synthetic datasets. The results indicate that our method provides improved signal detection in cases where sedimentation-rate changes and noise distortions are present.

The combination of convex optimization and the Yule-Walker AR model is capable of identifying the Milankovitch cycles present in the stratigraphic record. This algorithm not only strengthens the Milankovitch signal through spectral analysis, but it also minimizes noise, which is not a feature of the Milankovitch cycles’ periodicity. However, the selection of initial parameters, such as z and λ, becomes a crucial step in the success of this algorithm.

Selecting a 99% confidence level for spectral analysis strongly corresponds to identifying significant Milankovitch signals, providing high certainty that detected signals are not merely noise. This level is particularly advantageous for confirmatory studies or practical applications where the accuracy and reliability of results are paramount. However, the trade-off is a potentially broader confidence interval, which may reduce the precision of pinpointing exact frequencies. For initial exploratory studies, a lower confidence level, such as 95% or 90%, might be preferable, allowing for the identification of a wider range of potential signals at the risk of including more false positives. As a result, the choice of confidence level should be carefully considered in light of the study’s specific goals and context.

## Electronic supplementary material

Below is the link to the electronic supplementary material.

Supplementary Material 1

## Data Availability

All lithology code generated in this study is derived from published stratigraphic data. We provide the lithology code as Supplementary Material. The stratigraphic data is sourced from: https://doi.org/10.1016/j.palaeo.2005.11.004 (Molluscan bed section); https://doi.org/10.1016/j.epsl.2017.04.015 (the Mahakam Delta section of stratigraphy); https://doi.org/10.1007/s10933-020-00171-9 (Towuti Lake sediment section of stratigraphy); https://doi.org/10.17014/ijog.5.3.221-234 (Hominin fossil-bearing section, Flores).
